# Etymologia: Trombiculiasis

**DOI:** 10.3201/eid2604.ET2604

**Published:** 2020-04

**Authors:** Ronnie Henry

**Keywords:** trombiculiasis, infestation, mites, chiggers, parasites, Trombicula batatas

## Trombiculiasis [trom-bikʺu-liʹǝ-sis]

Infestation with mites of the family *Trombiculidae* (from the Greek *tromein*, “tremble,” and Latin *culex*, “gnat”) in their larval form (chiggers, from the Carib *chico*). A wide variety of livestock and wild animals, as well as humans, can become infested with chiggers (Figure). Trombiculid mites are vectors of *Orientia tsutsugamushi*, which causes scrub typhus. References to these mites appear as early as the sixth century in China. Linnaeus described the species *Trombicula batatas* in 1758.

**Figure:**
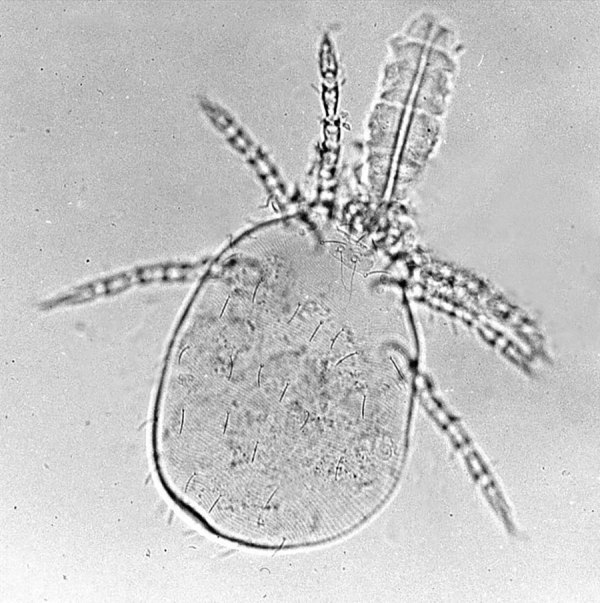
Photograph of a parasitic mite of domestic animals. Wikimedia Commons, Alan R. Walker, 2014.
